# Evaluation of ß-lactamase-induced inactivation of penicillin G and cefoperazone in spiked ultra-high temperature and raw unpasteurized milk

**DOI:** 10.3389/fvets.2026.1808217

**Published:** 2026-06-11

**Authors:** Martina Ludewig, Clair L. Firth, Michael Roschitz, Sophie H. Hinterleithner, Annemarie Käsbohrer

**Affiliations:** 1 Clinical Department for Farm Animals and Food System Transformation, Centre for Food Science, University of Veterinary Medicine Vienna, Vienna, Austria; 2 Clinical Department for Farm Animals and Food System Transformation, Centre for Veterinary Public Health & One Health, University of Veterinary Medicine Vienna, Vienna, Austria; 3 Department for Biological Safety, Federal Institute for Risk Assessment, Berlin, Germany

**Keywords:** antibiotics, enzymes, microbiological counts, pH, ß-lactamase, waste milk

## Abstract

**Introduction:**

In Austria, veterinary medicinal products (VMPs) containing penicillin G or cefoperazone are frequently used to treat dairy cows, which leads to the production of non-saleable waste milk. This study aimed to determine the loss of detectable antimicrobial activity of penicillin G and cefoperazone using a commercial ß-lactamase preparation, Antipen®.

**Methods:**

Ultra-high-temperature (UHT) processed and raw unpasteurized milk were spiked with penicillin G and cefoperazone (both pure pharmaceutical substances and VMPs), and the effects of temperature (37 °C, 25 °C, and 10 °C), storage time, and pH on Antipen® treatment on antibiotic activity were investigated. The aerobic mesophilic count (AMC) and *Enterobacteriaceae* before and after enzyme treatment in raw milk were also examined to assess the microbiological quality of milk that is potentially fed to calves.

**Results:**

The VMPs containing penicillin G (Vanapen®) or cefoperazone (Peracef®) in 50 mL of UHT or raw milk showed a higher stability after Antipen® treatment compared to pure antibiotic substances. Concentrations of 300 μg/mL of Vanapen® tested negative by Delvotest® T under all experimental conditions (12- and 6-h treatment, at natural pH and pH 5.5, at 37–10 °C). Treatments of milk with natural pH spiked with 10 μg/mL of Peracef® tested negative for the antibiotic after 12 and 6 h at 37 °C, while after acidification of the milk to pH 5.5, 10 μg/mL Peracef® was undetectable at all temperatures and time periods. In milk with natural pH treated with Antipen® for 12 h at 10–37 °C, the values of AMC always exceeded 5.0-log cfu/mL, indicating that the milk is not suitable for use as calf feed without prior acidification or subsequent pasteurization. Reducing the exposure time to 6 h or acidifying the milk to a pH of 5.5 during 12-h treatment significantly reduced bacterial growth.

**Discussion:**

This study demonstrated that Antipen® can effectively degrade penicillin, although its efficacy is lower and slower on cephalosporins. Since the study was conducted in a laboratory setting, extrapolation of the effective treatment durations to on-farm application requires validation in practical, real-world conditions.

## 1 Introduction

On dairy farms, antibiotics are frequently used to treat systemic and udder-associated diseases. Mastitis treatment is the most common indication for antibiotic use in dairy cows, with ß-lactam antibiotics–penicillin (systemic and intramammary) and third-generation cephalosporins (intramammary)–frequently used in Austria (1, 2). Following the administration of medicinal products (such as antibiotics) during lactation, waste milk that cannot be sold is produced. During the treatment of lactating cows with medicinal products and within the licensed withdrawal period, the milk produced is classified as waste milk and is unfit for human consumption due to potential drug residues (3, 4). However, research has shown a controversial association between this practice and negative health effects, including issues related to antibiotic resistance (5, 6). As summarized in a scientific opinion published by the European Food Safety Authority (EFSA), the selection pressure for antibiotic resistance in gut microbiota and the possible risk of resistance genes or residues being released into the environment are of significant concern from a One Health perspective (7). Several studies reported that feeding waste milk had similar or positive effects on daily weight gain, health, or performance of dairy calves compared with bulk milk or milk replacer (8–10). On the other hand, the practice of feeding waste milk and the number of antibiotic applications appear to correlate with the prevalence of extended-spectrum ß-lactamase-(ESBL)-producing *Escherichia coli* on farms (11) and led to a higher proportion of antibiotic resistant *E. coli* in calf feces compared to those fed bulk milk or milk replacer (3, 8, 12). Unpasteurized waste milk may contain significantly higher counts of aerobic mesophiles than pasteurized or bulk milk and may pose a risk for transmission of potential pathogens (8, 13, 14). Furthermore, it has been associated with an increased likelihood of diarrhea and a reduction in fecal microbiota diversity in pre-weaned calves, with a shift toward potential pathogens (15, 16). A few studies showed that even low concentrations of antibiotic residues in waste milk promote the development of resistance to those drugs in the calf’s intestine, which might negatively affect the health of animals (9, 17, 18). For this reason, it is important to investigate the degradation of antibiotic residues in waste milk. Various methods such as inactivation by heat, the use of ß-lactamase enzymes, microbial fermentation, and acidification or alkalinization of waste milk have been tested. Several studies indicated that penicillin and cephalosporins are relatively heat-stable in milk (19–22). Inactivation of antimicrobial residues was observed only at high temperatures and long exposure times, for example, penicillin: 4.0 μg/kg at 95 °C for 120 min and cefoperazone: 5.0 μg/kg at 120 °C for 20 min (19, 22). ß-lactamase enzymes are effective in decreasing penicillin residues in milk within 2–8 h, whereas the reduction of cephalosporin residues takes 24 h (22–24). Furthermore, adjusting the natural milk pH to lower (4.0–5.5) or higher (9.0–10.0) values can significantly increase the effectiveness of thermal activity in reducing residues (21, 22, 25, 26).

In the present study, the effect of temperature, storage times, and pH of treatments with the commercially available enzyme preparation Antipen® was investigated at different concentrations of penicillin G and cefoperazone (both pure pharmaceutical substances and veterinary medicinal products, VMP) in (i) ultra-high temperature processed and (ii) raw unpasteurized milk using a microbial growth inhibition assay to assess the loss of antibiotic activity after enzyme treatment. The milk was spiked with high and low concentrations of antibiotics to determine the limits of ß-lactamase activity in laboratory-scale experiments. Additionally, for each experimental combination in raw milk, the counts of aerobic mesophiles and *Enterobacteriaceae* before and after enzyme treatment were examined to assess the microbiological quality of milk potentially fed to calves. The overall aim of the present study was to identify, through laboratory-scale experiments, the most effective treatment combinations for reducing or eliminating penicillin G and cefoperazone residues in milk. Cefoperazone, a third-generation cephalosporin, was included in the study, as it was, at the time, still approved and a frequently used intramammary therapeutic agent in Austria (27). With the introduction of new Austrian drug legislation, its use no longer licensed in the country, although it remains approved for this indication in other European Union Member states (28, 29).

## 2 Materials and methods

### 2.1 Antibiotics, ß-lactamase, and milk samples

In preliminary test series carried out in 1 mL of ultra-high-temperature (UHT) processed milk, the loss of detectable antimicrobial activity of the pure pharmaceutical substances penicillin G (PENG; procaine benzylpenicillin, pharmaceutical reference standard, Merck KGaA, Darmstadt, Germany) and cefoperazone (CEF; cefoperazone dihydrate, Vetranal, analytical standard, Merck KGaA) was analyzed. UHT milk was initially spiked with 2,500 μg/mL PENG and then diluted serially to a concentration of 0.0025 μg/mL. Initial CEF concentration was 200 or 100 μg/mL milk, diluted to 0.02 or 0.01 μg/mL.

Further laboratory-scale experiments were performed with the veterinary medicinal products (VMPs) Vanapen® (VAN; active substance: benzylpenicillin procaine monohydrate, 300 mg/mL, intended for intramuscular use; Vana GmbH, Vienna, Austria) and Peracef® (PER; active substance: cefoperazone, 10 mg/mL, intended for intramammary use; Zoetis Deutschland GmbH, Berlin, Germany) in 50 mL of UHT or raw milk. The initial spiking concentration for VAN was 3,000 μg/mL, serially diluted to 0.003 μg/mL; for PER, it was 100 μg/mL, diluted to 0.01 μg/mL.

For the degradation of these antibiotic substances in milk, Antipen® (Vetcare Oy, Helsinki, Finland) was used, which is a commercially available β-lactamase developed for the degradation of penicillin G, ampicillin, and amoxicillin in waste milk. Antipen® is marketed in Finland, where veterinary antibiotic treatment guidelines primarily recommend penicillin for mastitis in dairy cattle (30). In addition to ß-lactamase, Antipen® also contains a potassium phosphate buffer, methyl parahydroxybenzoate, glycerin, and a food colorant additive to dye the treated milk blue. Experiments were conducted according to the manufacturer’s instructions with a volume of 25 μL/L. As Antipen® has not been evaluated for the degradation of cephalosporins, CEF concentrations in the range of 200–2.0 μg/mL were tested with both 25 and 250 μL Antipen®/L of milk.

The pH of the milk was adjusted with apple cider vinegar (5% acetic acid; standardized according to Codex Alimentarius Austriacus), as commonly applied in calf milk feeding, and measured with a Hanna pH meter (Professional pH & Temperature Meter HI98165, Hanna Instruments Inc., Woonsocket, United States) (31).

UHT milk (containing 3.5% fat and 3.3% protein) was purchased from retail markets, and raw unpasteurized milk (containing natural fat content 3.8–3.9%; and 3.4% protein, milked on the day of sampling) was collected directly from a milk filling station at a local farm. Before each experiment, the pH levels were carefully monitored, remaining between 6.5 and 6.7 in both UHT and raw milk, consistent with the natural pH (NpH) of fresh milk. Raw milk was stored at 5.0 °C for 12 to 24 h before being used for analysis.

### 2.2 Detection of antibiotic residues

The commercial broad-spectrum antibiotic test kit Delvotest® T, ampoules format, (DSM Firmenrich. Delft, Netherlands) with *Geobacillus stearothermophilus* var. *calidolactis* as an indicator organism, was used to assess the antimicrobial activity of antibiotic residues in UHT and raw milk following treatment with the β-lactamase preparation Antipen®. Delvotest® T is used as a screening method for the qualitative detection of antibiotic residues in milk in accordance with Implementing Regulation (EU) 2021/808 and enables detection of the maximum residue limits defined in Commission Regulation (EU) No 37/2010 (4 μg/kg for PENG and 50 μg/kg for CEF) (32, 33). The method has also been evaluated by the Association française de normalization (AFNOR), with detection limits of 0.003 μg for PENG and 0.02 μg for CEF per 1 mL of milk. In Austria, the use of Delvotest® T for residue control in milk is strongly recommended in accordance with the national legislation (34). Delvotest® T allows the simultaneous analysis of a large number of samples and was therefore selected as the method of choice in this study.

Before the experiments, the test system was carefully assessed. UHT milk was spiked with antibiotic substances (such as pure substances in 1 mL milk or VMPs in 50 mL milk) at initial concentrations and then diluted to the respective detection limits. The system was first verified in milk with NpH to ensure compliance with the required detection limits and was subsequently assessed in milk with experimentally modified pH values (6.0 and 5.5), followed by neutralization with 1.0 M sodium hydroxide solution (Merck KGaA), to evaluate performance under altered matrix conditions.

The UHT or raw milk batches were tested for the absence of antibiotic residues using Delvotest® T prior to each assay. An unspiked UHT or raw milk sample was used as a blank, in accordance with the manufacturer’s instructions, to determine the correct incubation time for the test. Furthermore, a control sample containing the final enzyme concentration and adjusted to the corresponding pH level was included in each trial. For subsequent experiments in acidified milk, the pH of the control and sample solutions was readjusted to 6.6 using 1.0 M sodium hydroxide before use in the detection assay. After completion of the predefined enzymatic incubation time, all samples were placed in crushed ice until inoculation of the ampoules with milk samples to halt further reactions.

In samples where the antibiotic concentration had degraded below the detection limit of the assay, growth of the test organism was indicated by a color change from purple to yellow. This color change was compared to the manufacturer’s reference scale and used for final evaluation.

### 2.3 Microbiological quantification of raw unpasteurized milk

Milk samples were mixed thoroughly, and the initial homogenate was serial decimal diluted (to 10^−8^) in Ringer’s solution (B. Braun, Melsungen, Germany) according to ISO 6887-1:2017 (35). Analyses of aerobic mesophilic counts (AMC) were performed according to ISO 4833-2:2013/Amd 1:2022 (36) on Trypto–Casein–Soy agar medium with 0.6% yeast extract (TSAYE, Biokar, Groupe Solabia, Pantin Cedex, France). *Enterobacteriaceae* (EB) were enumerated on Brilliance *E. coli*/Coliform Selective Agar (BECA, Thermo Fisher Scientific Inc., Oxoid, Waltham, MA, United States). Typical colony morphologies (1–3 isolates) for EB (colorless), coliforms (pink), and *E. coli* (purple) were selected and sub-cultivated on TSAYE for further confirmation by catalase (3%; Merck KgaA) and cytochrome-oxidase testing (BioMérieux, Marcy-l’Etoile, Frankreich). For the final *E. coli* confirmation, the ability to split indole from tryptophan was tested with the BactiDrop Spot Indol test on BECA (Thermo Fisher Scientific Inc., Oxoid). Counts for aerobic mesophiles, EB, and *E. coli* were calculated according to ISO 7218:2024 (37). The mean increase of AMC was calculated by the difference between the initial AMC of the untreated milk and the mean of the final AMC, comprising the samples with 300 μg/mL VAN, 10 μg/mL PER, and the control milk after all treatment procedures. The results were assigned to three categories, including “low” (<0.5 log Cfu/mL), “moderate” (0.5–1.5 log Cfu/mL), and “high” (>1.5 log Cfu/mL) increase, consistent with the assessment of microbial growth for shelf-life determination as outlined in the EFSA guidelines (38).

### 2.4 Statistics

Descriptive statistics (such as mean, standard deviation, and minimum and maximum values) of microbial counts were performed using Prism version 11.0.0 software for Windows (GraphPad Software, Boston, Massachusetts, United States, https://www.graphpad.com/). Logarithmically transformed microbial counts resulted in normally distributed data, which were assessed using the Kolmogorov–Smirnov test. A two-way analysis of variance (ANOVA) was conducted to assess the effects of storage temperature and the experimental conditions (such as time and pH) on microbial counts. In case of significant interactions, *post hoc* comparisons of simple effects were performed using the Tukey’s test. A *p*-value of <0.05 was considered statistically significant.

### 2.5 Experimental design

The experimental program of the study is illustrated in Table 1. The model matrix for waste milk was UHT or raw unpasteurized milk spiked either with a pure antibiotic substance or an antibiotic VMP. The initial spiking concentrations of the antibiotics were selected to approximate the respective therapeutic dose levels when referenced to 1 L of milk and were then serially diluted to the estimated detection limits. The preliminary tests evaluated the effectiveness of ß-lactamase Antipen® to inactivate the two pure antibiotic substances, PENG or CEF, in 1 mL UHT milk under different treatment conditions (such as storage temperature and duration, and pH of the milk). In further laboratory-scale experiments, the effectiveness of Antipen® for the inactivation of commercial preparations of veterinary antibiotic preparations containing PENG (VAN) or CEF (PER) was tested in 50 mL UHT and raw milk each. Parallel to experiments on antibiotic activity in unpasteurized raw milk, the microbiological counts for AMC and EB were determined in the control milk samples and milk spiked with VAN (300 μg/mL) or PER (10 μg/mL).

**Table 1:** Experimental program–degradation of antibiotics by ß-lactamase enzymes in UHT and raw unpasteurized milk.

Preliminary tests: degradation of pure pharmaceutical substances by Antipen® in 1 mL UHT milk		Laboratory-scale experiments: degradation of veterinary medical products by Antipen® in 50 mL UHT^b^ or unpasteurized raw milk	
Storage temperatures: 37, 25, and 10 °C			
Penicillin G^a^	Cefoperazone^a^	Vanapen®^a^	Peracef®^a^
12 h, NpH (1)	12 h, NpH (2)	12 h, NpH (4)	12 h, NpH (4)
12 h, pH 6.0 (1)	12 h, pH 5.5 (3)	12 h, pH 5.5 (4)	12 h, pH 5.5 (4)
12 h, pH 5.5 (1)	10 h, pH 5.5 (2)	6 h, NpH (3)	6 h, NpH (3)
6 h, NpH (3)	6 h, NpH (1)	6 h, pH 5.5 (3)	6 h, pH 5.5 (3)
4 h, NpH (2)	4 h, NpH (1)		
2 h, NpH (3)			

UHT: ultra-high temperature processed, NpH: natural pH of milk,

a Storage time, pH (number of replicates).

b A single series of analytical tests was carried out using UHT milk.

## 3 Results

### 3.1 Preliminary tests on the activity of pure antibiotics in 1 mL of UHT milk using Antipen®

In all experiments, PENG at concentrations of 2,500–0.025 μg/mL, treated with 0.025 μL Antipen® in 1 mL of UHT milk, tested negative in the Delvotest® T after 6 and 12 h at NpH, pH 6.0, and pH 5.5, and at 10, 25, and 37 °C. At shorter storage periods (4 and 2 h), 2,500 μg/mL PENG could not be consistently reduced below the detection limit (Table 2A). Concentrations of 200 μg/mL CEF were reduced by Antipen® (0.25 μL/mL) after 12 h (NpH and pH 5.5) and after 6 h (NpH), at 37 °C (Table 2B). CEF concentrations of 100 μg/mL were not detectable in UHT milk stored at 25 and 37 °C, at pH 5.5 for 10 h. In UHT milk spiked with lower CEF concentrations (20–0.1 μg/mL), Antipen® (0.25 μL/mL) reduced the antimicrobial substance to below the detection limit of Delvotest® T in all experiments (Table 2B).

**Table 2:** Degradation of (A) penicillin G (PENG) and (B) cefoperazone (CEF) in 1 mL spiked UHT milk by Antipen®.

(A) Storage temperature °C		10	25	37	10	25	37	10	25	37
Antipen® μL/mL	PENG μg/mL	12 h, NpH^a^			12 h, pH 6.0^a^			12 h, pH 5.5^a^		
0.025	2,500	**0/1**	**0/1**	**0/1**	**0/1**	**0/1**	**0/1**	**0/1**	**0/1**	**0/1**
	250	**0/1**	**0/1**	**0/1**	**0/1**	**0/1**	**0/1**	**0/1**	**0/1**	**0/1**
	25	**0/1**	**0/1**	**0/1**	**0/1**	**0/1**	**0/1**	**0/1**	**0/1**	**0/1**
	2.5	**0/1**	**0/1**	**0/1**	**0/1**	**0/1**	**0/1**	**0/1**	**0/1**	**1/1**
	0.25	**0/1**	**0/1**	**0/1**	**0/1**	**0/1**	**0/1**	**0/1**	**0/1**	**0/1**
	0.025	**0/1**	**0/1**	**0/1**	**0/1**	**0/1**	**0/1**	**0/1**	**0/1**	**0/1**
		6 h, NpH^a^			4 h, NpH^a^			2 h, NpH^a^		
0.025	2,500	**0/3**	**0/3**	**0/3**	1/2	1/2	**0/2**	2/3	2/3	2/3
	250	**0/3**	**0/3**	**0/3**	**0/2**	**0/2**	**0/2**	**0/3**	**0/3**	**0/3**
	25	**0/3**	**0/3**	**0/3**	**0/2**	**0/2**	**0/2**	**0/3**	**0/3**	**0/3**
	2.5	**0/3**	**0/3**	**0/3**	**0/2**	**0/2**	**0/2**	**0/3**	**0/3**	**0/3**

(B) Storage temperature °C		10	25	37	10	25	37	10	25	37
Antipen® μL/mL	CEF μg/mL	12 h, NpH^a^			12 h, pH 5.5^a^			6 h, NpH^a^		
0.25	200	2/2	1/2	**0/2**	2/3	2/3	**0/3**	1/1	1/1	**0/1**
	20	**0/2**	**0/2**	**0/2**	**0/3**	**0/3**	**0/3**	**0/1**	**0/1**	**0/1**
	2	**0/2**	**0/2**	**0/2**	**0/3**	**0/3**	**0/3**	**0/1**	**0/1**	**0/1**
	0.2	**0/2**	**0/2**	**0/2**	**0/3**	**0/3**	**0/3**	**0/1**	**0/1**	**0/1**
		10 h, pH 5.5^a^			4 h, NpH^a^					
0.25	100	2/2	**0/2**	**0/2**	1/1	1/1	**0/1**			
	10	**0/2**	**0/2**	**0/2**	**0/1**	**0/1**	**0/1**			
	1	**0/2**	**0/2**	**0/2**	**0/1**	**0/1**	**0/1**			
	0.1	**0/2**	**0/2**	**0/2**	**0/1**	**0/1**	**0/1**			

a Number of samples positive for antibiotics/number of assayed by Delvotest® T. Degraded samples are highlighted in bold. Limit of detection: PENG 0.0025/CEF 0.02 μg/mL.

Milk stored at: 10 °C, 25 °C, and 37 °C (A) for 12 h, at natural pH (NpH) and pH 6.0 and 5.5, and for 6 h, 4 h, and 2 h, at NpH and (B) for 12 h, 6 h, and 4 h, at natural pH (NpH), for 12 h and 10 h, at pH 5.5.

### 3.2 Activity of antibiotic drugs in 50 mL of UHT and raw unpasteurized milk using Antipen®

In laboratory-scale experiments, the effects of Antipen® (0.025 μL/mL for VAN and 0.25 μL/mL for PER) on the stability of VAN and PER in 50 mL of milk were evaluated under different conditions. Samples were stored for 6 or 12 h at NpH or pH 5.5 and at 10, 25, or 37 °C. The results are presented in Tables 3A,B. High PER concentrations (100 μg/mL) remained detectable using Delvotest® T under all tested conditions. In contrast, high VAN concentrations (3,000 μg/mL) were no longer detectable after Antipen® treatment within 12 h at 37 °C (NpH) and at 25 °C (pH 5.5). Lower VAN concentrations (300–0.03 μg/mL) showed no detectable activity under any experimental conditions (Table 3A). Treatments of milk (NpH) spiked with a lower concentration of PER (10 μg/mL) tested negative using Delvotest® T after 12 and 6 h at 37 °C. After acidification of the milk to pH 5.5, PER at 10 μg/mL was consistently undetectable (Table 3B).

**Table 3:** Degradation of (A) Vanapen® (VAN) and (B) Peracef® (PER) in 50 mL spiked UHT or raw unpasteurized milk by Antipen®.

(A) Storage temperature °C		10	25	37	10	25	37	10	25	37	10	25	37
Antipen® μL/mL	VAN μg/mL	12 h, NpH^a^			12 h, pH 5.5^a^			6 h, pH 5.5^a^			6 h, pH 5.5^a^		
0.025	3,000	4/4	3/4	**0/4**	3/4	**0/4**	1/4	3/3	3/3	3/3	3/3	3/3	3/3
	300	**0/4**	**0/4**	**0/4**	**0/4**	**0/4**	**0/4**	**0/3**	**0/3**	**0/3**	**0/3**	**0/3**	**0/3**
	30	**0/4**	**0/4**	**0/4**	**0/4**	**0/4**	**0/4**	**0/3**	**0/3**	**0/3**	**0/3**	**0/3**	**0/3**
	3	**0/4**	**0/4**	**0/4**	**0/4**	**0/4**	**0/4**	**0/3**	**0/3**	**0/3**	**0/3**	**0/3**	**0/3**
	0.3	**0/4**	**0/4**	**0/4**	**0/4**	**0/4**	**0/4**	**0/3**	**0/3**	**0/3**	**0/3**	**0/3**	**0/3**
	0.03	**0/4**	**0/4**	**0/4**	**0/4**	**0/4**	**0/4**	**0/3**	**0/3**	**0/3**	**0/3**	**0/3**	**0/3**

(B) Storage temperature °C		10	25	37	10	25	37	10	25	37	10	25	37
Antipen® μL/mL	PER μg/mL	12 h, NpH^a^			12 h, pH 5.5^a^			6 h, pH 5.5^a^			6 h, pH 5.5^a^		
0.25	100	4/4	3/4	**0/4**	3/4	**0/4**	1/4	3/3	3/3	3/3	3/3	3/3	3/3
	10	3/4	2/4	**0/4**	**0/4**	**0/4**	**0/4**	3/3	1/3	**0/3**	**0/3**	**0/3**	**0/3**
	1	**0/4**	**0/4**	**0/4**	**0/4**	**0/4**	**0/4**	**0/3**	**0/3**	**0/3**	**0/3**	**0/3**	**0/3**
	0.1	**0/4**	**0/4**	**0/4**	**0/4**	**0/4**	**0/4**	**0/3**	**0/3**	**0/3**	**0/3**	**0/3**	**0/3**

a Number of samples positive for antibiotics/number assayed by Delvotest® T. Degraded samples are highlighted in bold. Limit of detection: VAN 0.003/PER 0.01 μg/mL.

Milk stored at: 10 °C, 25 °C, and 37 °C for 12 h and 6 h, at natural pH (NpH) and pH 5.5.

### 3.3 Microbiological quantification in raw unpasteurized milk during storage experiments with Antipen®

**Figure 1:**
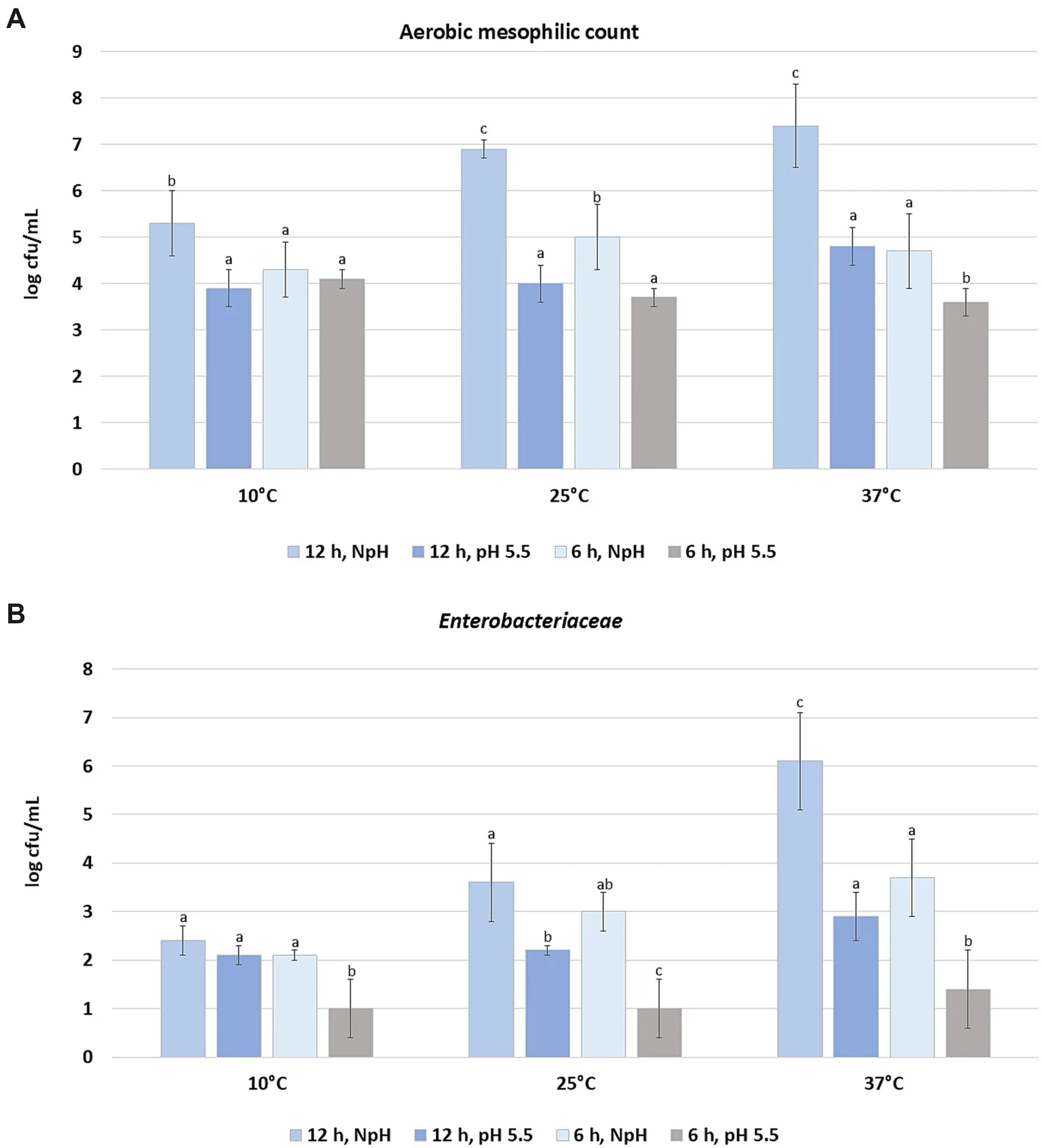
Mean microbiological counts ± standard deviation of **(A)** aerobic mesophilic count (AMC) and **(B)** *Enterobacteriaceae* in raw unpasteurized milk treated with Antipen® after 12 and 6 h of storage at 10 °C, 25 °C, and 37 °C, at natural pH (NpH) and pH 5.5. The mean was calculated for each experimental combination based on counts in the control sample and milk spiked with Vanapen® (VAN 300 μg/mL) or Peracef® (PER 10 μg/mL). Different subscript letters above the bars indicate statistically significant differences among experimental conditions within each temperature group (*p* < 0.05).

In the untreated raw milk, the initial AMC ranged from 2.6 to 4.9 log cfu/mL, and counts of EB were between 1.0 and 3.0 log cfu/mL. The mean AMC obtained in raw milk without and with antibiotics (VAN 300 μg/mL or PER 10 μg/mL) after 12 h of treatment with Antipen® at NpH was in the range of 5.3 cfu/mL at 10 °C and 7.4 log cfu/mL at 37 °C (*p* < 0.05), while in milk at pH 5.5 the mean AMC was estimated to be between 3.9 log cfu/mL at 10 °C and 4.8 log cfu/mL at 37 °C (*p* < 0.05; Supplementary Table 1). After 6 h of storage, the raw milk samples treated with Antipen® showed a mean AMC of 4.3 (10 °C) to 4.7 log cfu/mL (37 °C) at NpH and 4.1 (10 °C) to 3.6 log cfu/mL (37 °C) at pH 5.5 (*p* > 0.05; Supplementary Table 1). In general, the mean counts of EB were lower than those of AMC in all experiments. The highest microbial load (6.1 log cfu/mL) was found in raw milk (NpH) stored for 12 h at 37 °C, and the lowest loads (0.7–2.3 log cfu/mL) were observed with the experimental combination 6 h and pH 5.5 at 10, 25, and 37 °C (*p* < 0.05; Figure 1B, Supplementary Table 1). Higher counts (such as 5.3 and 3.3 log cfu/mL) of potential *E. coli* were only determined with the combination 37 °C, 12 and 6 h, NpH (Supplementary Table 1). The analysis showed that the time/pH combinations influenced the mean count of AMC and *Enterobacteriaceae* in a temperature-dependent manner and that the magnitude of the temperature effect varied among the experimental conditions (*p* < 0.05; Figures 1A,B).

**Figure 2:**
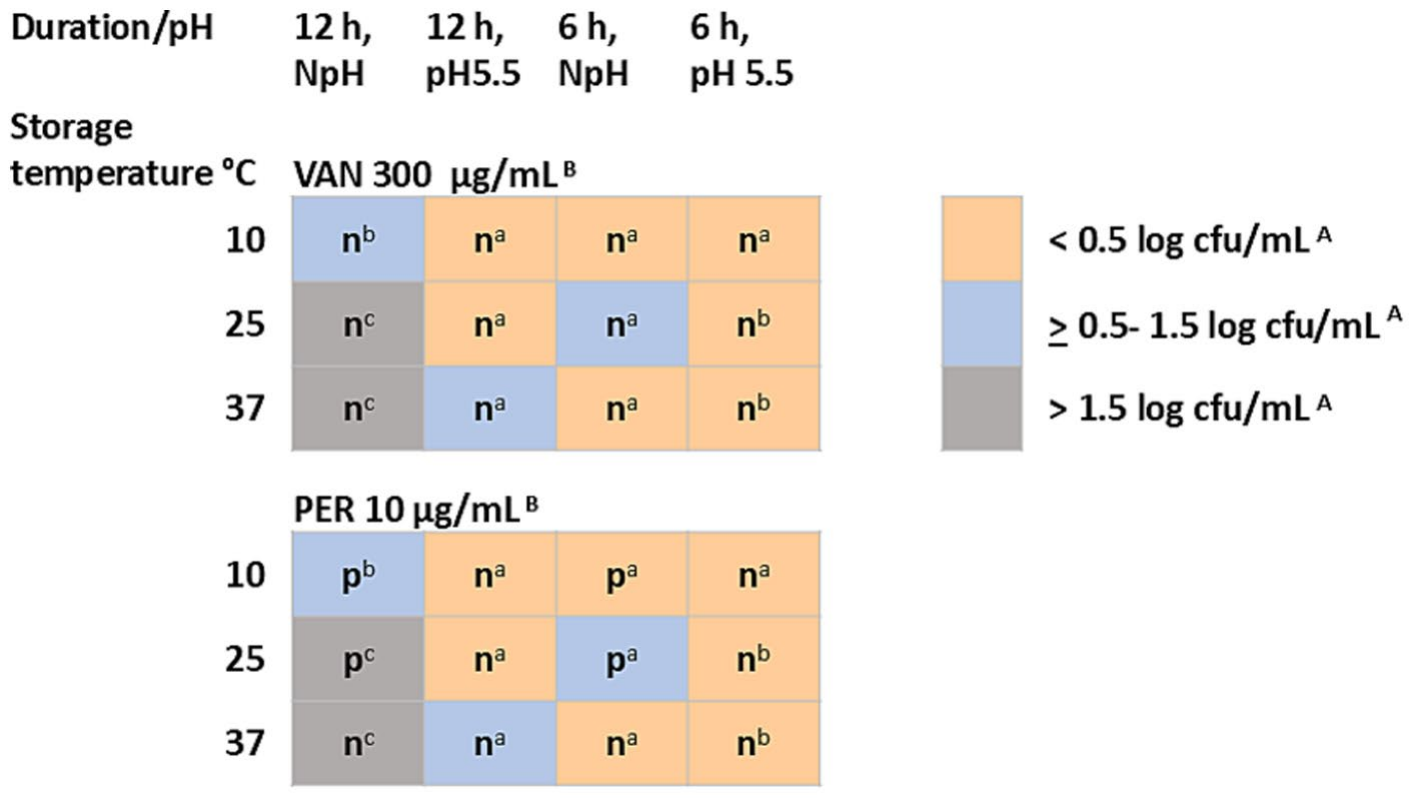
Heat maps illustrating the mean increase of the aerobic mesophilic count (AMC) in raw unpasteurized milk by category^A^ after 12 and 6 h of storage at 10 °C, 25 °C, and 37 °C, at natural pH (NpH), pH 5.5, and treated with Antipen®. The increase was calculated by the difference between the initial AMC in untreated milk and the mean of the final AMC comprising samples with Vanapen® (VAN 300 μg/mL), Peracef® (PER 10 μg/mL), and the control milk. ^B^p/n—milk positive/negative for antibiotics assayed by Delvotest® T. Different subscript letters in a cell indicate statistically significant differences in AMC increase among experimental conditions for each temperature (*p* < 0.05).

The microbiological increase in raw milk treated with Antipen® stored for 12 and 6 h at different temperatures and pH values is illustrated in Figure 2, with the results of detectable antibiotic residues assayed by Delvotest® T. In general, the AMC increase was significantly influenced by the interaction between temperature and the time/pH combination (*p* < 0.05; Figure 2). The AMC found in milk after enzyme treatment for 12 h (NpH) at 25 and 37 °C was high (>5.0 log cfu/mL) and increased by more than 1.5 log cfu/mL. The AMC increase for the other test combinations was “moderate” (≥0.5–1.5 log cfu/mL) to “low” (<0.5 log cfu/mL), with a high final AMC (>5.0 log cfu/mL) in all or single samples at combination: 10 °C, 12 h (NpH); 25 °C, 6 h (NpH); and 37 °C, 12 h (pH 5.5) and 6 h (NpH) (Figure 2, Supplementary Table 1). High EB counts (>5.0 log cfu/mL) with an increase of more than 1.5 log cfu/mL were only observed after treatment at 37 °C, 12 h (NpH). The categories “low” to “moderate” increases in AMC correspond to VAN levels (300 μg/mL) below the detection limit after 12 h (pH 5.5) and 6 h (NpH, pH 5.5) and PER (10 μg/mL) after 12 and 6 h (pH 5.5) at 10, 25, and 37 °C. However, PER was still detectable in samples after treatment at 10 and 25 °C for 6 h, at NpH.

## 4 Discussion

The study aimed to identify a combination of treatment conditions suitable for practical use in farms to decrease residue concentrations in waste milk, focusing on two important antibiotic substances, including penicillin G and cefoperazone (PENG and CEF), frequently used in Austrian mastitis therapy. Antibiotics of the ß-lactam family are characterized by the ß-lactam ring in their chemical structure and include aminopenicillins and cephalosporins. These antibiotics can be inactivated by the hydrolysis of ß-lactamase enzymes, which break the ring open (39).

The results of preliminary tests in 1 mL of UHT milk showed that the commercially available β-lactamase Antipen®, at the concentration recommended by the manufacturer (25 μL/L), reduced high levels of pure PENG (2,500 μg/mL) within 6–12 h and over a broad temperature range from 10 to 37 °C below the detection limit of Delvotest® T.

Furthermore, the acidification of milk to pH of 6.0 and 5.5 for 12 h had no additional effect on detectable PENG residues by Antipen®. The highest concentration (200 μg/mL) of pure CEF could only be inactivated at 37 °C in 1 mL UHT milk with NpH within 6 and 12 h and in acidified milk after 12 h. However, as Antipen® is not evaluated to inactivate cephalosporins, it is not surprising that a 10-fold dose of the enzyme was required to degrade CEF. Previous studies (22, 23) reported complete ß-lactamase degradation of PENG (1 μg/mL and 25 μg/g) within 2 and 8 h at 20–37 °C. The fourth-generation cephalosporin, cefquinome (2 μg/mL) was eliminated after 24 h at 10 °C (24). However, it is important to note that these studies investigated the inactivation of lower antibiotic concentrations compared to this study.

This study observed that experiments with the VMPs (VAN, active antibiotic substance PENG and PER, active antibiotic substance CEF) in 50 mL of raw unpasteurized or UHT milk showed a higher stability of these substances after treatment with Antipen® compared to pure antibiotics. One possible explanation is the excipients used in these formulations: povidone in VAN, which exhibits binding and stabilizing properties, and peanut oil in PER, which reduces solubility (40, 41).

Furthermore, CEF was more stable in all experiments, and both the pure substance and the corresponding drug were less reduced by ß-lactamase than PENG. This was expected, as cephalosporins are more resistant to opening their ß-lactam ring than PENG (39). Acidification of milk to pH 5.5 improved the reduction of both drugs compared to milk with NpH. High levels of the VMP VAN (PENG 3000 μg/mL) in acidified milk were reduced below the detection limit at 25 °C after 12 h with ß-lactamase, and PER (CEF) at 10 μg/mL was reliably reduced after 12 and 6 h treatment with Antipen® at pH 5.5 and 10–37 °C. In the study presented by Renner (42), ß-lactamase hydrolysis of PENG was extended in milk acidified to pH 5.0 and 5.5, and the enzyme was no longer active in milk with a pH below 4.5. However, Li et al. (23) only observed complete degradation of PENG by ß-lactamase in milk with NpH. For cephalosporins, comparable studies have not been published to date. Hydrolysis of cephalosporins in pure milk increased when the NpH was changed to an alkaline pH of 9.0–10.0 (21, 24, 25); however, the palatability of alkalinized milk to calves remains questionable (21, 24).

A few studies have previously determined that residue levels in waste milk from the same antibiotic can vary significantly. In waste milk from various farms, the maximum residue concentration ranged from 0.026–2.5 mg/L for PENG and 1.7–4.6 mg/L for cefquinome, a fourth-generation cephalosporin, and the maximum level reported for cefoperazone was 0.15 mg/kg (9, 18, 43). In the study conducted by Pereira et al. (44), waste milk contained most frequently PENG but in very low concentrations, with a mean level of 0.008 mg/L, and the third-generation cephalosporin (ceftiofur; mean 0.15 mg/L). It has also been noted that milk on the first day after systemic administration contains a very low concentration of PENG (0.092 mg/L), and within 7 days it decreased below the assigned maximum residue level of 4 μg/kg (45). PENG was eliminated from milk below this MRL within 72–96 h (depending on milking frequency) after three intramammary treatments (46). Cefoperazone (limit of detection 0.002 μg/mL, MRL 50 μg/kg in milk) was detectable between 60 and 84 h following a single intramammary administration (47). Both the injectable VMP VAN and the intramammary formulation PER used in this study had a legal milk withdrawal period in the EU of 5 days after the last treatment (48, 49).

It is also important to note that the VMP for PENG, including VAN, was a solution for intramuscular injection in dairy cattle rather than an intramammary formulation, which would, therefore not necessarily be present in this form in the udder and/or milk. Comparable penicillin-containing products for intramammary injections do exist; however, these were not commercially available at the time of the study. The VMP containing CEF, namely PER, was an intramammary formulation, although it should still be considered that cows are not generally milked immediately after intramammary tube application, meaning that some pharmacokinetic changes would be expected *in vivo*.

The experimental findings in the laboratory suggest that a concentration of 300 μg/mL VAN (active antibiotic substance PENG) or 10 μg/mL PER (active antibiotic substance CEF) can be successfully reduced below the detection limit of Delvotest® T by the ß-lactamase Antipen® within a reasonable time period. Experimental pharmacokinetic studies provide indicative estimates of residual concentrations expected in waste milk; however, milking intervals and the applied dose and treatment intervals under experimental conditions do not always reflect those used in clinical practice. A study investigating the excretion of PENG reported a mean residual milk concentration of 103.5 μg/mL 12 h after a single intramammary administration of the therapeutic dose (50). For CEF, the calculated concentration excreted into milk 12 h after administration of a single high intramammary dose of 1,200 mg ranged between 47.7 and 525 μg/mL in cows with infected udders (47). However, in practice, these CEF concentrations are unlikely to be achieved, as it is not standard practice to treat all four quarters of the udder for mastitis, and 10 mL of PER contains only 100 mg of CEF for one application.

Based on reported residue levels and evaluating concentrations obtained in pharmacokinetic studies of both antibiotics, higher concentrations are not expected to occur in waste milk on farms, particularly since waste milk represents a mixture of milk collected at different withdrawal times.

Microbial quality is an extremely important aspect of waste milk fed to calves. The requirements for AMC in raw waste milk fed to calves should be the same as for raw unpasteurized milk used for human consumption according to Regulation (EC) No 853/2004 (51): The limit for this is 5.0 log cfu/mL, as also recommended by Godden et al. (52). High AMC poses a health risk for calves, as mastitis-causing bacteria or other potentially pathogenic bacteria (such as *Staphylococcus* spp., *Streptococcus* spp., *Listeria monocytogenes*, *Campylobacter* spp., Shiga toxin-producing *E. coli*, and *Salmonella* spp.) may occur in raw milk and could increase during unrefrigerated storage (14, 53–55).

In this study, milk with NpH treated with Antipen® for 12 h, at 10–37 °C showed AMC values exceeding the limit of 5.0 log cfu/mL. This long-period treatment in NpH milk can therefore not be recommended without additional pasteurization. In addition, very high EB and *E. coli* counts (>7.0 log cfu/mL) are to be expected at very high ambient temperatures (37 °C). Reducing the exposure time to 6 h, or alternatively acidifying the milk to pH 5.5 with a 12-h treatment, resulted in a significant reduction in bacterial growth (*p* < 0.05), with only a small number of individual samples reaching AMC above the specified limit. The initial bacterial load in waste milk is difficult to estimate, as it varies considerably dependent on mastitis pathogen and the cow’s immune response to infection. Values of 3.3 to 6.9 log cfu/mL have been reported in the literature, and higher counts may be associated with mastitis (8, 10, 14, 56).

The present study found that acidification of the milk with apple cider vinegar to pH 5.5, combined with a ß-lactamase treatment for 12 h at 10 and 25 °C, and for 6 h at 10–37 °C, resulted in an increase of AMC of less than 0.5 log cfu/mL (categorized as “low”). Acidified milk has long been known to prevent bacterial growth in normal and waste milk (57) and has also been reported to have positive effects on calf health status (16, 58).

Increases in microbial counts in waste milk are influenced by multiple factors, including intrinsic and extrinsic conditions (such as temperature, pH, and storage time), the initial bacterial load, variability in microbial growth optima, and variability in antibiotic residue concentrations. Concurrently, the values of 300 μg/mL VAN and 10 μg/mL PER could be reduced below the detection limit of the Delvotest® T by Antipen® treatment. The blue colorant of Antipen® prevents misuse and possible addition of treated waste milk into the food chain.

One limitation of this study is that Delvotest® T is a qualitative screening method and therefore does not provide quantitative information on antibiotic residue concentrations in milk. However, the test is validated by AFNOR, and the detection limits for PENG and CEF are below the MRLs assigned by the EU (32). A further limitation of this study is that milk acidification was performed using apple cider vinegar. Other commercially available acidifying agents may have more complex compositions, including additives such as sugars and dextrin, which could influence both the degradation of antibiotic residues and the increase of microbial count. These laboratory-scale experiments provide a basis for potential application on farms. Validation for larger milking volumes and practical farm conditions is still required.

## 5 Conclusion

Expected residue levels in waste milk depend on the antibiotic type, route of administration (such as systemic or intramammary), number of treatments, milking frequency, udder health, and post-treatment period. Overall, these results show that veterinary drugs containing penicillin or cefoperazone can be successfully reduced below the detectable antimicrobial activity in milk by ß-lactamases. The efficacy of the enzyme treatment is influenced by several factors, including exposure time, milk pH, and treatment temperature. These experiments with the VMPs, penicillin (VAN), and cefoperazone (PER), suggest that the exposure time to degrade residues of these two antibiotics should be at least 6 h. Acidifying the milk to pH 5.5 improved the inactivation process and prevented a large increase in microbial load in raw milk during enzyme treatment. If ß-lactamase treatment is performed without acidification, the milk should always be pasteurized after enzyme exposure; otherwise, bacterial growth may pose a risk to calf health.

## Data Availability

The raw data supporting the conclusions of this article will be made available by the authors, without undue reservation.

## Author’s note

The commercial enzyme, Antipen® was provided to the authors for research purposes at a reduced wholesale price by the Finnish company, VetCare Oy. However, employees of this company were not involved at any stage in the planning or execution of this experimental study, nor in the writing of this manuscript.
