# Correction: Evaluation of ß-lactamase-induced inactivation of penicillin G and cefoperazone in spiked ultra-high temperature and raw unpasteurized milk

**DOI:** 10.3389/fvets.2026.1981737

**Published:** 2026-09-16

**Authors:** Frontiers Production Office

**Affiliations:** Frontiers Media SA, Lausanne, Switzerland

**Keywords:** antibiotics, enzymes, microbiological counts, pH, ß-lactamase, waste milk

The funder University of Veterinary Medicine Vienna was erroneously omitted from the **Funding** statement. The correct Funding statement reads:

“The author(s) declared that financial support was received for this work and/or its publication. This study was conducted within the COMET-Project D4Dairy (Digitalisation, Data integration, Detection and Decision support in Dairying) that is supported by BMK (Austrian Federal Ministry of Climate Action, Environment, Energy, Mobility, Innovation and Technology), BMDW (Austrian Federal Ministry of Digital and Economic Affairs) and the provinces of Niederösterreich and Vienna in the framework of COMET-Competence Centers for Excellent Technologies. The COMET program is handled by the FFG (grant number 872039). Open access funding provided by University of Veterinary Medicine Vienna.”

The original version of this article has been updated.

