# Supplementary material for: Evaluation of ß-lactamase-induced inactivation of penicillin G and cefoperazone in spiked ultra-high temperature and raw unpasteurized milk
Source: Front Vet Sci. 2026 Jun 11;13:1808217. doi: 10.3389/fvets.2026.1808217 (PMC13305172; doi:10.3389/fvets.2026.1808217)
Supplement: Supplementary file 1 [file Table_1.DOCX]

**Supplementary Table 1** Mean microbiological counts in raw milk after 12 and 6 hours of storage at 10, 25 and 37°C, at natural pH (NpH) and pH 5.5. The mean was calculated for each experimental combination based on counts in the control milk and milk spiked with Vanapen® (VAN 300 µg/ml), Peracef® (PER 10 µg/ml).

| **Experimental combination** | **Aerobic mesophilic count** | | | | ***Enterobacteriaceae*** | | | ***Escherichia coli*** | | | |
| --- | --- | --- | --- | --- | --- | --- | --- | --- | --- | --- | --- |
|  | **Storage temperature °C** | | | | | | | | | | |
|  | **10** | **25** | **37** | **10** | | **25** | **37** | | **10** | **25** | **37** |
| **12 h, NpH** | 5.3 + 0.7^A,b^ | 6.9 + 0.2^a^ | 7.4 + 0.9^a^ | 2.4 + 0.3^b^ | | 3.6 + 0.8^a^ | 6.1 + 1.0^a^ | | 1.1 + 0.6 | 1.9 + 1.4 | 5.3 + 1.8 |
|  | (4.5; 6.5)^B^ | (6.4; 7.3) | (6.2; 9.0) | (2.0; 3.0) | | (2.0; 4.6) | (4.3; 7.9) | | (0.7; 2.3) | (0.7; 4.0) | (0.7; 7.8) |
| **12 h, pH 5.5** | 3.9 + 0.4^a^ | 4.0 + 0.4^a^ | 4.8 + 0.4^b^ | 2.1 + 0.2^a^ | | 2.2 + 0.1^a,b^ | 2.9 + 0.5^a^ | | 1.1 + 0.6 | 0.8 + 0.4 | 1.2 + 0.9 |
|  | (3.3; 4.5) | (3.5; 4.8) | (4.0; 5.6) | (1.7; 2.3) | | (2.0; 2.4) | (2.2; 3.4) | | (0.7; 2.3) | (0.7; 2.0) | (0.7; 3.4) |
| **6 h, NpH** | 4.3 + 0.6^a^ | 5.0 + 0.7^a^ | 4.7 + 0.8^a^ | 2.0 + 0.1^b^ | | 3.0 + 0.4^a^ | 3.7 + 0.8^a^ | | 0.7 + 0.0 | 1.2 + 1.0 | 3.3 + 1.3 |
|  | (3.5; 5.0) | (4.0; 6.0) | (3.3; 5.5) | (2.0; 2.3) | | (2.0; 3.5) | (2.3; 4.6) | |  | (0.7; 3.5) | (0.7; 4.6) |
| **6 h, pH 5.5** | 4.1 + 0.2^a^ | 3.7 + 0.2^a^ | 3.6 + 0.3^a^ | 1.0 + 0.6^a^ | | 1.0 + 0.6^a^ | 1.4 + 0.8^a^ | | 0.7 + 0.0 | 0.9 + 0.5 | 1.2 + 0.7 |
|  | (3.7; 4.4) | (3.3; 3.8) | (3.0; 4.0) | (0.7; 2.3) | | (0.7; 2.3) | (0.7; 2.3) | |  | (0.7; 2.0) | (0.7; 2.3) |

^A^mean ± SD; Different subscript letters indicate statistically significant differences in AMC and EB count among experimental conditions for each temperature (*p < 0.05*).^B^minimum; maximum given in log cfu/mL.; Counts of 0.7 cfu/g signifies value below the detection limit and used for the calculation of the mean.
